# Isolation and characterisation of lymphatic endothelial cells from lung tissues affected by lymphangioleiomyomatosis

**DOI:** 10.1038/s41598-021-88064-3

**Published:** 2021-04-16

**Authors:** Koichi Nishino, Yasuhiro Yoshimatsu, Tomoki Muramatsu, Yasuhito Sekimoto, Keiko Mitani, Etsuko Kobayashi, Shouichi Okamoto, Hiroki Ebana, Yoshinori Okada, Masatoshi Kurihara, Kenji Suzuki, Johji Inazawa, Kazuhisa Takahashi, Tetsuro Watabe, Kuniaki Seyama

**Affiliations:** 1 Division of Respiratory Medicine, Juntendo University Faculty of Medicine and Graduate School of Medicine, 3-1-3 Hongo; Bunkyoku, Tokyo, 113-8431 Japan; 2 Study Group for Pneumothorax and Cystic Lung Diseases, Tokyo, Japan; 3 Department of Biochemistry, Graduate School of Medical and Dental Sciences, Tokyo Medical and Dental University, Tokyo, Japan; 4 Division of Pharmacology, Graduate School of Medical and Dental Sciences, Niigata University, Niigata, Japan; 5 Department of Molecular Cytogenetics, Medical Research Institute, Tokyo Medical and Dental University, Tokyo, Japan; 6 Pneumothorax Research Center and Division of Thoracic Surgery, Nissan Tamagawa Hospital, Tokyo, Japan; 7 Department of Thoracic Surgery, Tokyo Metropolitan Bokutoh Hospital, Tokyo, Japan; 8 Department of Thoracic Surgery, Institute of Development, Aging and Cancer, Tohoku University, Sendai, Japan; 9 Department of General Thoracic Surgery, Juntendo University School of Medicine, Tokyo, Japan

**Keywords:** Biochemistry, Cell biology, Computational biology and bioinformatics, Diseases, Pathogenesis

## Abstract

Lymphangioleiomyomatosis (LAM) is a rare pulmonary disease characterised by the proliferation of smooth muscle-like cells (LAM cells), and an abundance of lymphatic vessels in LAM lesions. Studies reported that vascular endothelial growth factor-D (VEGF-D) secreted by LAM cells contributes to LAM-associated lymphangiogenesis, however, the precise mechanisms of lymphangiogenesis and characteristics of lymphatic endothelial cells (LECs) in LAM lesions have not yet been elucidated. In this study, human primary-cultured LECs were obtained both from LAM-affected lung tissues (LAM-LECs) and normal lung tissues (control LECs) using fluorescence-activated cell sorting (FACS). We found that LAM-LECs had significantly higher ability of proliferation and migration compared to control LECs. VEGF-D significantly promoted migration of LECs but not proliferation of LECs in vitro. cDNA microarray and FACS analysis revealed the expression of vascular endothelial growth factor receptor (VEGFR)-3 and integrin α9 were elevated in LAM-LECs. Inhibition of VEGFR-3 suppressed proliferation and migration of LECs, and blockade of integrin α9 reduced VEGF-D-induced migration of LECs. Our data uncovered the distinct features of LAM-associated LECs, increased proliferation and migration, which may be due to higher expression of VEGFR-3 and integrin α9. Furthermore, we also found VEGF-D/VEGFR-3 and VEGF-D/ integrin α9 signaling play an important role in LAM-associated lymphangiogenesis.

## Introduction

Lymphangioleiomyomatosis (LAM) is a rare lung disease that typically affects women of childbearing age, and is characterised by the proliferation of abnormal smooth muscle-like cells (LAM cells) which leads to diffuse cystic destruction of the lungs. LAM cells are low-grade metastasizing neoplasms harboring mutations in either the *TSC1* or *TSC2* gene, tumor suppressor genes encoding hamartin or tuberin, respectively. *TSC1* or *TSC2* mutations results in dysregulated mechanistic/mammalian target of rapamycin complex 1 (mTORC1) signaling in LAM cells^1^.

As expressed in the name of disease, lymphangiogenesis is the conspicuous pathological feature of LAM. LAM lesions in the lungs as well as retroperitoneal lymphangioleiomyomas, have abundant lymphatic vessels with irregularly dilated spaces or slit-like appearance together with proliferating LAM cells^2,3^. Corresponding with these pathological findings, LAM patients frequently develop lymphatic manifestations including chylous fluid accumulation in the pleural and/or peritoneal spaces, pulmonary lymphatic congestion, and lower extremity lymphedema^4–6^. In this context, LAM has been clinically recognized as a disease involving the lymphatic system^5^. Furthermore, LAM-associated lymphangiogenesis has been implicated in the spread of LAM cells and disease progression because it seems to mediate fragmentation of LAM lesions and shedding of LAM cells as LAM cell clusters (LCCs) into the lymphatic stream^3,7^. LCCs, globular aggregates of LAM cells enveloped by a monolayer of lymphatic endothelial cells (LECs), are histopathologically identified in the lymphatic vessels of LAM lesions including the lungs, lymphangioleiomyomas, and the uterus^2,3,7,8^. Additionally, LCCs are frequently found in various types of chylous effusion such as pleural effusion and ascites, a characteristic and pathognomonic complication found in 10–15% of LAM patients^7,9^. Identification of LCCs in chylous effusion and their cytopathological characterisation are valuable in diagnosing LAM^10^. Therefore, exploring the mechanisms for LAM-associated lymphangiogenesis likely identifies the therapeutic targets to control the disease progression of LAM.

LAM cells produce and secrete lymphangiogenic vascular endothelial growth factor-D (VEGF-D), which appears to play an important role in disease progression by recruiting LECs and promoting their proliferation^3,7,11^. VEGF-D is elevated in the blood of LAM patients whereas VEGF-A and -C are not^11^. Therapeutic intervention with rapamycin/sirolimus, a mechanistic/mammalian target of rapamycin (mTOR) inhibitor, successfully decreases serum VEGF-D levels as well as stabilizes of pulmonary function in LAM patients^12,13^. Therefore, VEGF-D and its receptors vascular endothelial growth factor receptor (VEGFR)-2 or -3 on LECs have been implicated as central mechanisms of LAM-associated lymphangiogenesis. However, few studies have focused on functional characteristics of LAM-associated LECs and the precise role of VEGF-D in LAM-associated lymphangiogenesis. In this study, we established a flow cytometry-based method to isolate LECs from LAM-affected lung tissues and analysed the biological features of LAM-associated LECs. This contributes to a better understanding of the pathobiology of LAM, a disease involving the lymphatic system.

## Results

### Isolation of LECs from lung tissues that are expandable in vitro

**Figure 1:**
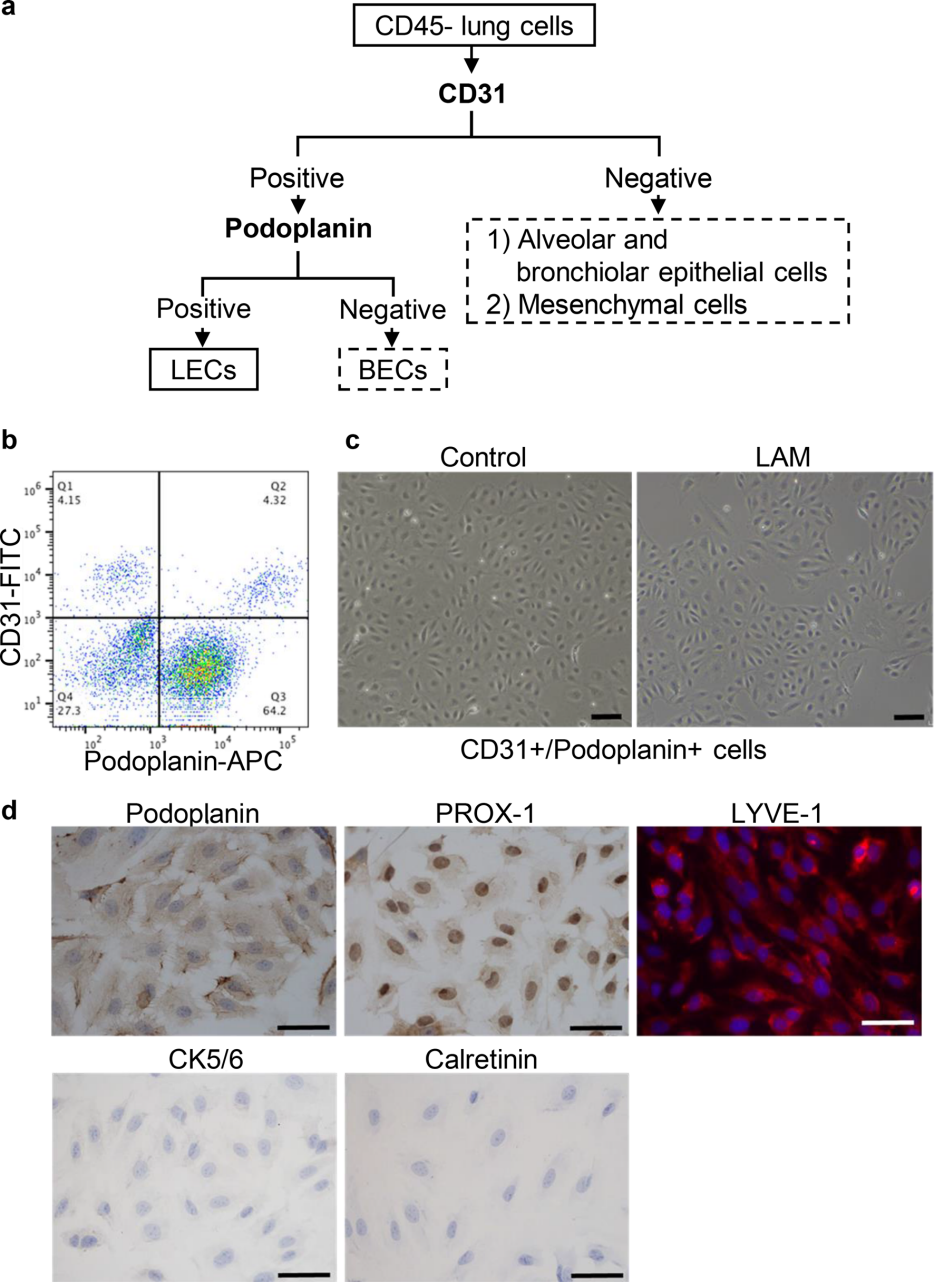
Isolation of LECs from lung tissues. (**a**) Schema of the method for isolating LECs from lung tissues. The boxes with dotted lines indicate the cell populations not pertinent to this study. (**b**) A representative FACS dot plot showing the expression of CD31 and podoplanin in cultured CD45- lung cells. (**c**) A representative morphology of cultured CD31+/podoplanin + cells (right panel is from LAM lung tissue; left panel is from control lung tissue) under an inverted microscope (scale bar = 100 µm). (**d**) Representative microphotographs of immunostaining to characterise cultured CD31+/podoplanin + cells (scale bar = 50 µm). They were immunocytochemically positive for anti-podoplanin and anti-PROX1 antibodies and negative for CK 5/6 and calretinin (DAB, i.e., brown pigment, was used as a chromogen). Immunofluorescence staining for LYVE1 revealed positive red cytoplasmic staining (nuclei were counterstained with DAPI).

Using flow cytometry, we obtained cells which highly express both CD31 and podoplanin from CD45-lung cells (Fig. 1a, b). These CD31+/podoplanin + cells grew with a cobblestone appearance, suggestive of an endothelial phenotype (Fig. 1c). To ensure isolated CD31+/podoplanin + cells were LECs, we examined expression of the lymphatic-specific cell markers podoplanin, prospero homeobox protein 1 (PROX-1) and lymphatic vessel endothelial hyaluronic acid receptor 1 (LYVE-1) with immunocytochemistry and immunofluorescence staining. Additionally, we examined the expression of both cytokeratin 5/6 (CK5/6) and calretinin in these CD31+/podoplanin + cells to exclude possible contamination of podoplain-positive mesothelial cells. As shown in Fig. 1d, cultured CD31+/podoplanin + cells consistently expressed podoplanin, PROX1 and LYVE-1; more than 97% of cells were positively immunostained by each antibody. Conversely, they were negative for CK5/6 and calretinin. These results indicated that our CD31+/podoplanin + cells possessed the distinct phenotypic characteristics of LECs.

LECs were isolated from explanted LAM lung tissues from LAM patients (*n* = 4), video-assisted thoracoscopic surgery (VATS)-derived LAM lung tissues (*n* = 3), and normal parts of lung tissue from lung cancer patients (*n* = 4). Table 1 shows the yields and viability of LECs by type of lung tissue. Hereafter, we will designate these types of LECs as LAM-LECs (T), LAM-LECs (V), and control LECs, respectively. The yield of LECs in 1 × 10^6^ of CD45- lung cells across all tissue types was 1.98 ± 1.63 × 10^4^ (mean ± SD), and LECs’ viability as examined by the dye-exclusion method was 87.4% ± 6.3 (mean ± SD).

**Table 1:** Lung tissues utilized to isolate LECs with yields and viability of LECs.

Case No	Age/gender	Lung tissue	Tissue weight (g)	No. of LECs per CD45-cells (1.0 × 10^6^)	Viability (%)
1	57/F	Normal	3.8	1.7 × 10^4^	81
2	56/F	Normal	2.1	3.2 × 10^4^	91
3	63/F	Normal	1.0	0.8 × 10^4^	85
4	57/F	Normal	2.3	0.7 × 10^4^	96
5	26/F	LAM (VATS)	1.7	1.2 × 10^4^	80
6	30/F	LAM (VATS)	3.5	0.4 × 10^4^	94
7	44/F	LAM (VATS)	3.2	2.2 × 10^4^	89
8	48/F	LAM (Transplantation)	20.1	1.6 × 10^4^	84
9	62/F	LAM (Transplantation)	41.3	3.8 × 10^4^	77
10	41/F	LAM (Transplantation)	19.0	0.5 × 10^4^	93
11	45/F	LAM (Transplantation)	33.9	5.6 × 10^4^	91

*LAM* lymphangioleiomyomatosis, *LECs* lymphatic endothelial cells, *VATS* video-assisted thoracoscopic surgery.

### LAM-LECs (T) showed a greater ability of proliferation

**Figure 2:**
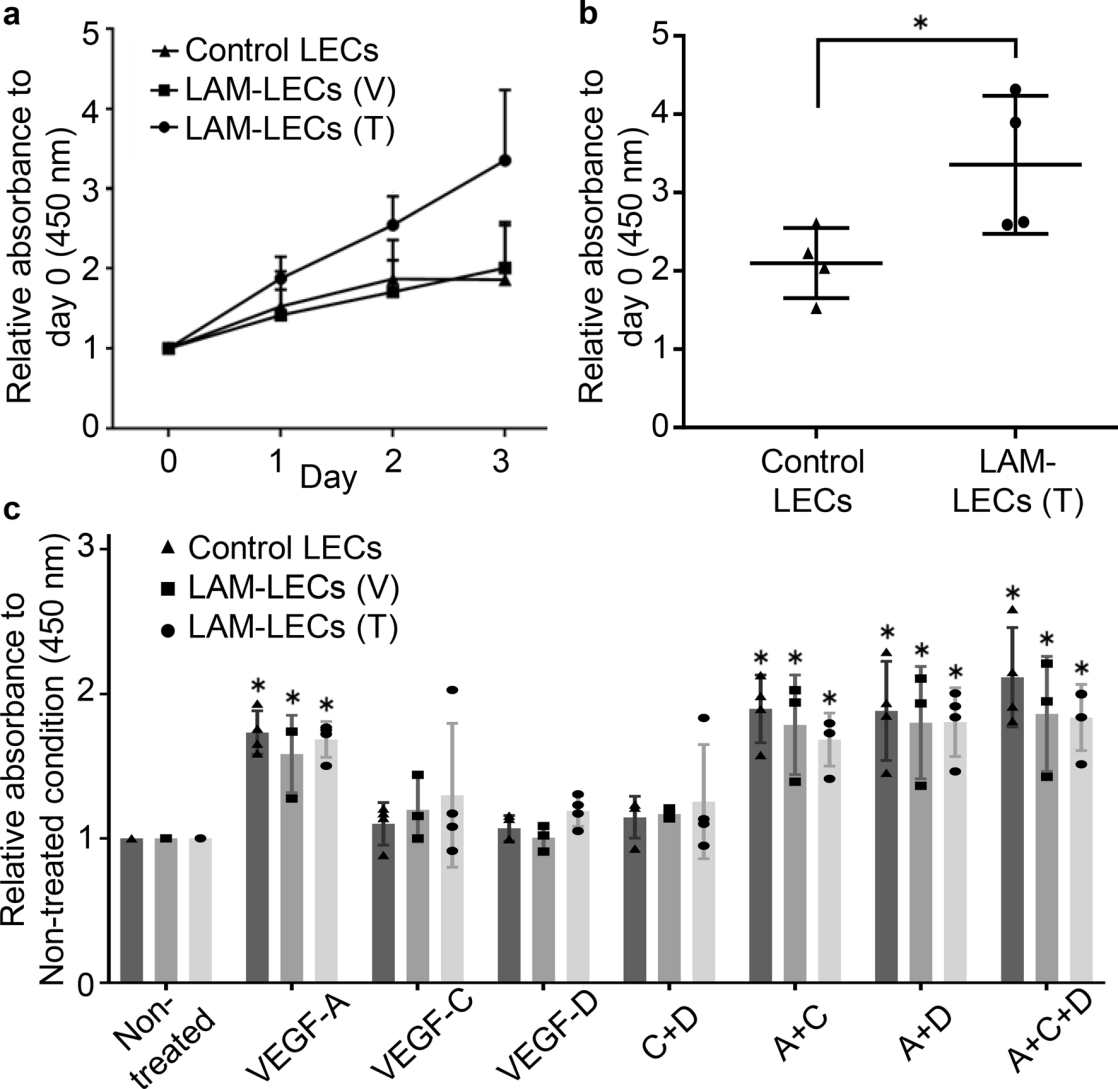
Proliferative characteristics of LECs isolated from lung tissues. (**a, b**) Plots of proliferation when cultured in ECBM/5% FBS. (**a**) Cell growth was examined for 3 days and is expressed on the vertical axis as the relative ratio of absorbance (450 nm) compared with that on Day 0. LAM-LECs (T) (*n* = 4), LAM-LECs (V) (*n* = 3), and control LECs (*n* = 4). (**b**) Individual results of LAM-LECs (T) and control LECs at Day 3 were plotted. (**c**) Plot of proliferative responses for various growth factors. LECs were cultured in ECBM/5% FBS and stimulated for 3 days with VEGF-A (10 ng/mL), -C (50 ng/mL), -D (10 ng/mL), or a combination of 2–3 growth factors. Growth response is expressed on the vertical axis as the relative ratio of absorbance (450 nm) compared with that of each type of non-treated data (i.e., absorbance without growth factors). LAM-LECs (T) (*n* = 4), LAM-LECs (V) (*n* = 3), and control LECs (*n* = 4). Statistical significance was assessed using the Student’s t-test and one-way ANOVA followed by Tukey's multiple comparisons tests. *p < 0.05.

We compared the ability of each type of LEC to proliferate in Endothelial Cell Basal Medium ([ECBM]; PromoCell)/5% FBS. As shown in Figs. 2a and 2b, LAM-LECs (T) proliferated significantly faster than control LECs at Days 3. Conversely, LAM-LECs (V) showed no difference in proliferation compared with control LECs. Next, we evaluated the effects of various growth factors on proliferation of LECs (Fig. 2c). Compared to the control condition (i.e., ECBM/5% FBS without growth factors), only VEGF-A significantly promoted the proliferation of LECs. In contrast, neither VEGF-C nor -D promoted LECs’ growth to the same degree. When LECs were co-stimulated with VEGF-A and either VEGF-C, -D or the combination of all 3 growth factors, we found no additive effect of either VEGF-C, -D, or both, on the degree of proliferation elevated by VEGF-A alone. Furthermore, we did not find any difference among LAM-LECs (T), LAM-LECs (V), or control LECs in the responses to VEGF-A, -C, -D, or their combinations.

### LAM-LECs (T) showed greater ability to migrate

**Figure 3:**
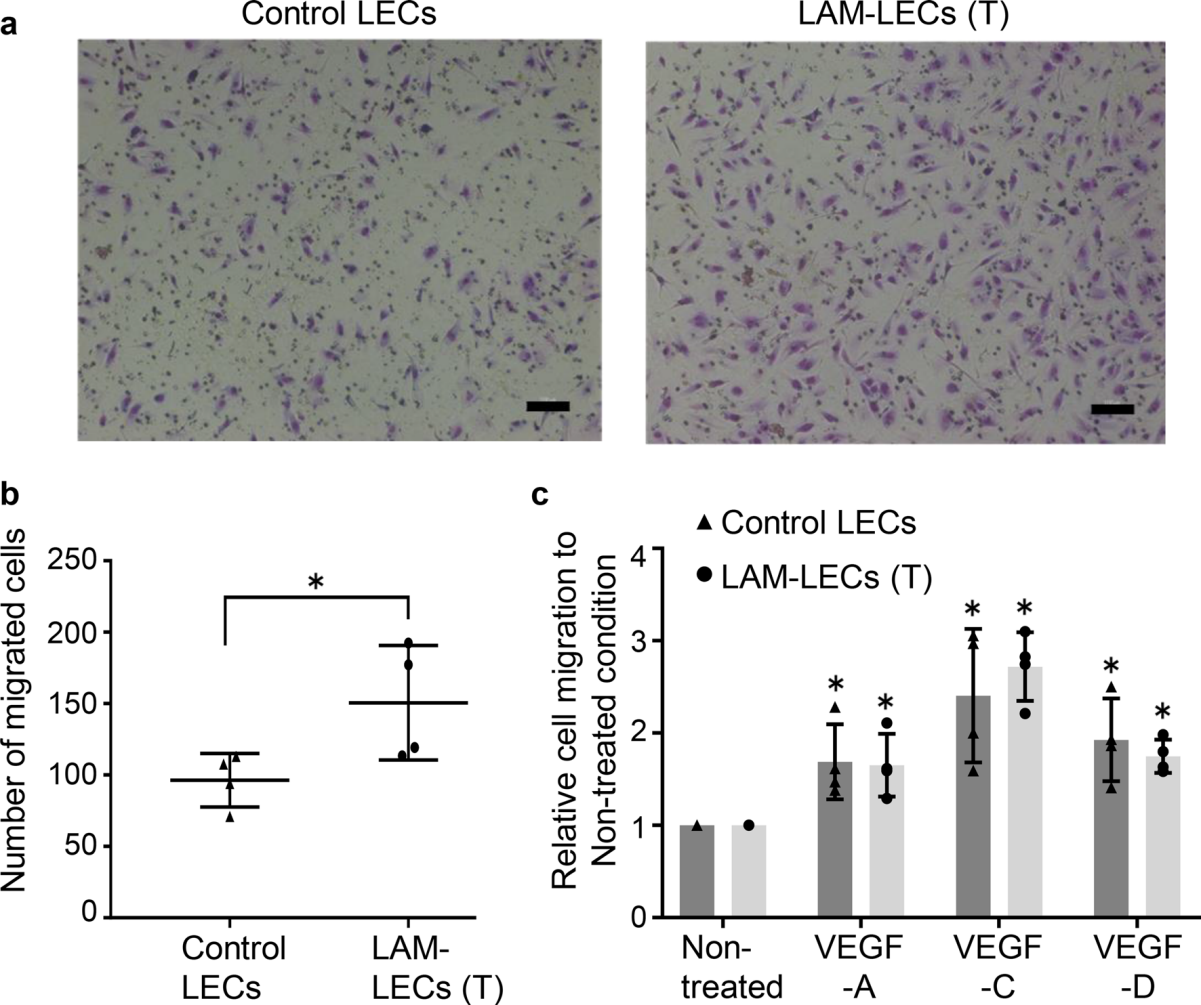
Chamber migration assay of LECs isolated from lung tissues. (**a**) Representative microphotographs of LECs migrated to the lower side of membranes after incubation for 4 h. Membranes were stained with Diff-Quik (nuclei were stained as purple). (**b**) Plot of results from migration assay of LECs. LAM-LECs (T) (*n* = 4) and control LECs (*n* = 4). (**c**) Plot graph showing migration of LECs when stimulated by various growth factors. Chamber migration assays were performed with the addition of VEGF-A (10 ng/mL), -C (50 ng/mL), or -D (100 ng/mL). Responses are expressed on vertical axis as relative ratios of migrated cells compared with those of non-treated data (i.e., the number of migrated cells without added growth factors). LAM-LECs (T) (*n* = 4) and control LECs (*n* = 4). Statistical significance was assessed using the Student’s t-test and one-way ANOVA followed by Tukey's multiple comparisons test. *p < 0.05.

To evaluate the ability of LECs to migrate, we performed a chamber migration assay (Fig. 3a). We found that LAM-LECs (T) showed significantly higher mobility than that of control LECs when incubated in ECBM/0.5% FBS (Fig. 3b). Next, we analyzed the effect of VEGF-A, -C, and -D on the migration of LECs (Fig. 3c). All of the growth factors we tested significantly promoted LECs’ migration, with the greatest effect exerted by VEGF-C stimulation when compared to the control condition (i.e., ECBM/0.5% FBS without growth factors). VEGF-A, -C, and -D significantly promoted the migration of LECs, respectively, compared with the control condition (i.e., ECBM with 0.5% FBS without growth factors). However, no significant difference in migratory response to any of these growth factors was found between LAM-LECs (T) and control LECs.

### Complementary DNA (cDNA) microarray analysis revealed LAM-LECs (T)-specific pathways and gene sets

**Figure 4:**
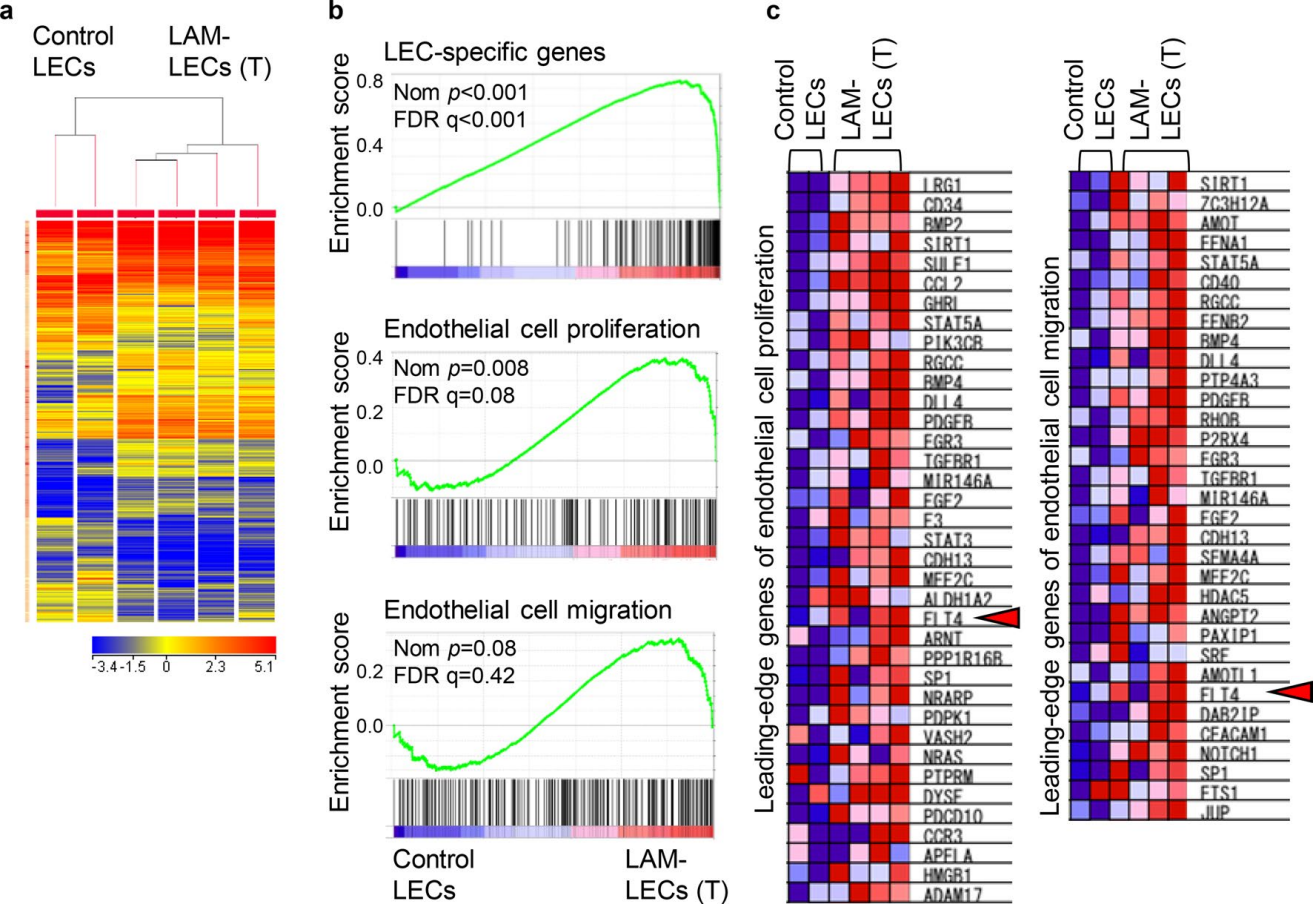
cDNA microarray analysis of LAM-LECs and control LECs. (**a**) Tree-view analysis of the average-linkage hierarchically clustered gene expression data. Genes in which mRNA expression changes 2 or more-fold between LAM-LECs (T) and control LECs are shown. The top dendrogram represents the similarity between individual arrayed samples (vertical plane) based on the global gene expression profile. (**b**) GSEA of LAM-LECs (T) compared to control LECs using gene sets included in the LEC-specific gene signatures, GO terms “endothelial cell proliferation” and “endothelial cell migration”. A nominal p‐value (Nom *p*) < 0.01 and a false discovery rate (FDR) < 0.25 were considered as statistically significant. (**c**) The heat map shows genes comprising the leading edge of the GSEA plot. Red indicates high expression; blue indicates low expression. Note that *FLT4* encoding VEGFR-3 is identified as one of highly expressed genes in LAM-LECs (T) (arrowheads).

To identify genes regulating the phenotypical characteristics of LAM-LECs (T), we performed a cDNA microarray. cDNA was obtained from 4 samples of LAM-LECs (T) and 2 samples from control LECs. A total of 1281 genes had more than two-fold changes in the expression between the LAM-LECs (T) and control LECs groups (Fig. 4a). The pathway analysis using these genes elucidated the genes included in focal adhesion, and the phosphoinositide 3-kinase (PI3K)-AKT signaling pathways were significantly enriched in LAM-LECs (T) (Table 2). A total of 37 genes was involved in these pathways (Supplementary Tables S3 and S4). Notably, gene set enrichment analysis (GSEA) revealed that LEC-specific genes were significantly enriched in LAM-LECs (T) (Fig. 4b)^14^. In addition, genes of the Gene Ontology (GO) term “endothelial cell proliferation” were significantly enriched in LAM-LECs (T) compared to control LECs (Fig. 4b).

**Table 2:** The top 15 significant pathways enriched in LAM-LECs (T).

Pathway	*p* value
Focal adhesion	< 1.00 × 10^–10^
PI3K-AKT signaling pathway	< 1.00 × 10^–10^
Myometrial relaxation and contraction pathways	< 1.00 × 10^–10^
Vitamin D receptor pathway	< 1.00 × 10^–10^
Focal adhesion–PI3K–AKT–mTOR-signaling pathway	< 1.00 × 10^–10^
Spinal cord injury	3.01 × 10^–10^
Human complement system	5.74 × 10^–10^
Arrhythmogenic right ventricular cardiomyopathy	3.98 × 10^–9^
VEGF-A/VEGFR-2 signaling pathway	9.36 × 10^–9^
MAPK signaling pathway	2.52 × 10^–8^
Hippo-Merlin signaling dysregulation	5.91 × 10^–8^
Nuclear receptors meta-pathway	7.85 × 10^–8^
Complement and coagulation cascades	2.37 × 10^–7^
Hair follicle development cytodifferentiation	2.93 × 10^–7^
miRNA targets in ECM and membrane receptors	7.94 × 10^–7^

The top 15 pathways enriched in LAM-LECs (T) analysed by GeneSpring software are listed.

**Figure 5:**
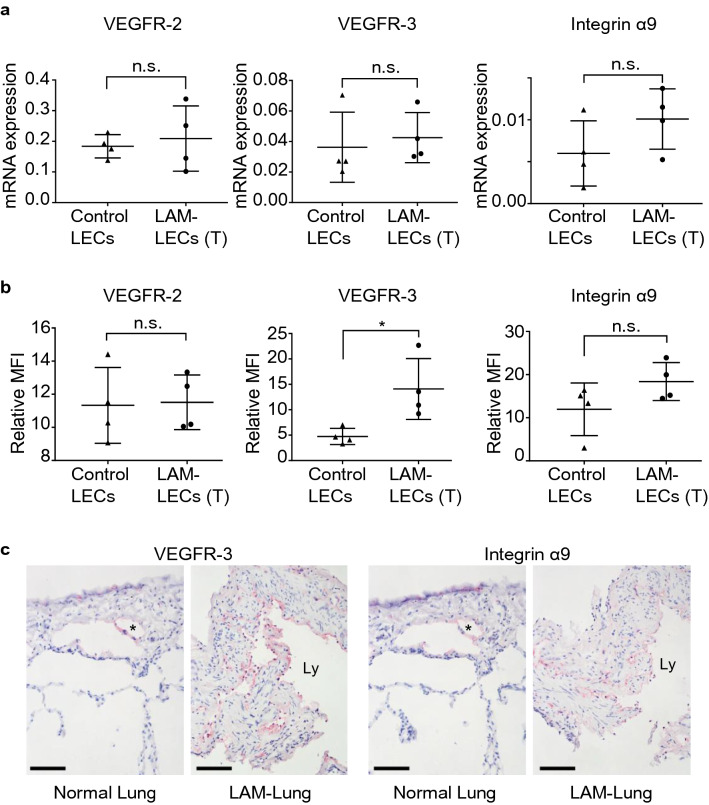
The expression of VEGFR-2, VEGFR-3 and integrin α9 by LECs. (**a**) Validation of cDNA microarray analysis with quantitative RT-PCR. Plots of the relative mRNA expression of VEGFR-2, VEGFR-3, and integrin α9 to GAPDH. LAM-LECs (T) (n = 4) and control LECs (n = 4) were examined. (**b**) Plots of the expression of VEGFR-2, VEGFR-3, and integrin α9 analysed as the MFI measured by FACS. The relative MFI of each sample to isotype control is expressed on the vertical axis. LAM-LECs (T) (n = 4) and control LECs (n = 4) were examined. (**c**) Images of immunohistochemistry of lung tissues showing expression of VEGFR-3 and integrin α9 (Fast red was used as a chromogen, scale bar = 200 µm). In normal lung tissue, lymphatic vessels (*) surrounded by a single-layer of LECs are seen in the visceral pleura. In LAM-lungs, proliferation of spindle-shaped LAM cells and irregularly dilated lymphatic vessels (Ly) lined by a monolayer of LECs are indicated. Note that LECs were immunopositive for VEGFR-3 and integrin α9 antibodies in both normal and LAM-lungs. Statistical significance was assessed using the Student’s t-test. *p < 0.05 and n.s. = not significant.

Among the genes listed in the leading-edge genes (Fig. 4c and Supplementary Table S5) as well as the focal adhesion and PI3K-AKT signaling pathways (Table 2), we especially focused on *ITGA9* and *FLT4* which encode integrin α9 and VEGFR-3, respectively, because these function as receptors for VEGF-D, a key lymphangiogenic molecule in LAM. To verify the transcriptional changes detected by cDNA microarray analysis, quantitative real-time reverse transcription polymerase chain reaction ([PCR]; qRT-PCR) regarding genes encoding VEGFR-2, -3 and integrin α9 was performed (Fig. 5a). To further examine the expression of these genes in protein levels, we performed flow cytometry of LAM-LECs (T) and control LECs for VEGFR-2, -3, and integrin α9. VEGFR-3 expression significantly increased in LAM-LECs (T) compared to control LECs. Integrin α9 was also expressed higher in LAM-LECs (T) than control LECs, however, it was not statistically significant in protein levels (Fig. 5b). We also confirmed the expression of both VEGFR-3 and integrin α9 in LECs for LAM-lung and normal lung tissues obtained from patients. As shown in Fig. 5c, we found that LECs were immunopositive for VEGFR-3 and integrin α9 antibodies in both normal and LAM-lungs.

### VEGFR-3 is involved in both proliferation and migration of LAM-LECs (T) whereas integrin α9 participates in VEGF-D-mediated migration of LAM-LECs (T)

**Figure 6:**
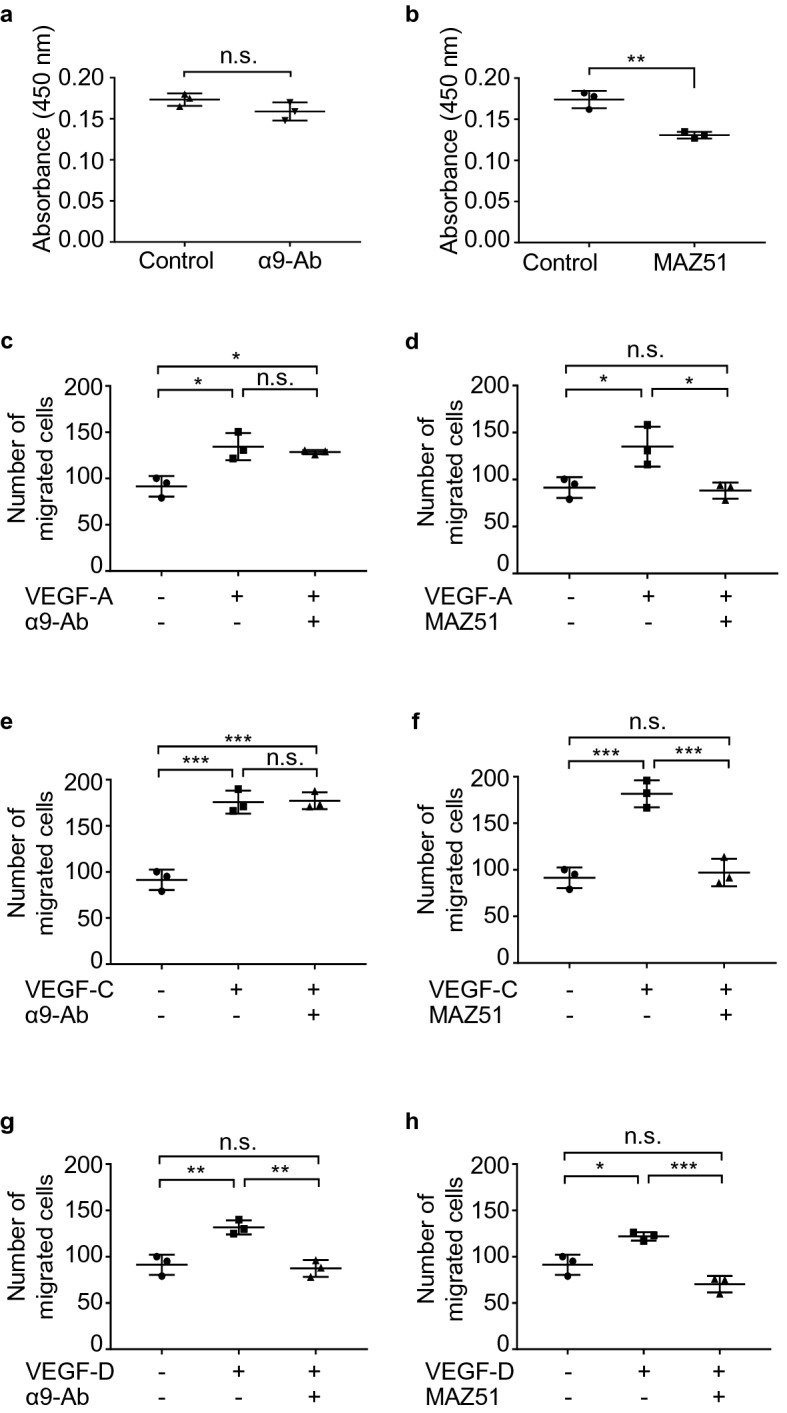
The role of VEGFR-3 and integrin α9 in proliferation and migration of LECs. (**a, b**) Dot plot graphs of the effects of MAZ51 and the anti-integrin α9-neutralizing antibody (α9-Ab) on proliferation of LAM-LECs (T). LAM-LECs (T) were cultured in ECBM with 5% FBS for 3 days with the anti-integrin α9-neutralizing antibody (50 μg/mL) (**a**) or with MAZ51 (10 μM) (**b**). Growth response is expressed on the vertical axis as the relative ratio of absorbance (450 nm). (**c-h**) Dot plot graphs of the effects of MAZ51 and the anti- integrin α9-neutralizing antibody on migration of LAM-LECs (T). Chamber migration assays of LECs were performed using various grow factors: (**c, d**) VEGF-A (10 ng/mL); (**e, f**) VEGF-C (50 ng/mL); and (**g, h**) VEGF-D (100 ng/mL). Statistical significance was assessed using the Student’s t-test (**a, b**) or one-way ANOVA followed by Tukey’s multicomparison test (**c-h**). *p < 0.05, ** *p* < 0.01, *** *p* < 0.001 and n.s. = not significant. Abbreviations: α9-Ab = anti-integrin α9 neutralizing antibody.

We also evaluated the effect of VEGFR-3 kinase inhibitor (MAZ51) and the anti-integrin α9-neutralizing antibody on proliferation and migration of LECs using a representative sample of LAM-LECs (T). MAZ51 significantly suppressed the proliferation of LAM-LECs (T) while the anti-integrin α9-neutralizing antibody did not (Fig. 6a, b). In the migration assay, the migration of LAM-LECs (T) promoted by VEGF-A, -C, and -D were significantly inhibited by MAZ51 (Fig. 6d, f, h). Notably, we found that the anti-integrin α9-neutralizing antibody specifically suppressed only the effect of VEGF-D, but not VEGF-A and -C (Fig. 6c, e, g).

## Discussion

To the best of our knowledge, this is the first report to reveal the biological characteristics of LAM-associated LECs. We found that LAM-LECs (T) have a higher ability to proliferate and migrate partly due to the higher expression of VEGFR-3 and integrin α9. Additionally, VEGF-D appears to have a greater role in promoting migration than proliferation via interaction with integrin α9.

First, we established a flow cytometry-based method to obtain LECs with high purity from LAM-affected lungs as well as normal lung tissues. In our previous report, we directly sorted LECs from CD45- lung cells by flow cytometry immediately after the preparation of single cell suspension^15^. To that end, we used multiple antibodies with different fluorochromes to separate LECs from other lung cell populations, e.g., vascular endothelial cells, bronchial and alveolar epithelial cells, and mesenchymal cells. However, though isolated LECs were viable, we frequently experienced that LECs did not attach onto collagen I-coated plates for several weeks after seeding, and LECs stopped proliferating within 1 or 2 passages. We speculated that damage to the cells, possibly due to successive protease digestion, multiple antibodies binding to cell surfaces, laser irradiation to cells, and other similar processes would be the cause. Therefore, in this study we included the step of in vitro co-culture of CD45-lung cells on the collagen I-coated plates for approximately 1 week to let them recover from possible damage before sorting LECs by flow cytometry. This modified protocol enabled us to obtain a higher number of purified LECs than was possible with our previous method. Furthermore, isolated LECs were functionally competent; LECs immediately attached onto collagen I-coated plates and continued proliferation for at least 8 passages with distinct lymphatic phenotypes.

The exact mechanisms by which our modified protocol succeeded to isolate and culture LECs from human lung tissues remain undetermined. Some studies have reported that LECs were isolated from human cancer tissues. All these studies included the process of co-culturing LECs with other tissue-composing cells for a certain period until cells attached onto culture dishes and started proliferation^16–18^. Accordingly, we speculate that the process of culturing LECs with other CD45- lung cells may play an important role. This method may mimic in vivo cell–cell interactions of each lung-composing cell, enabling isolated LECs to keep their biological activities in vitro.

In this study, LECs isolated from LAM-affected lungs are likely to include at least 2 types of LECs: LAM-associated LECs occupying the abundant lymphatic vessels in LAM lesions and LECs existing in normal parts of the lungs aside from LAM lesions. The characteristics of LAM-LECs (T) strongly indicate that the phenotype of LAM-associated LECs differs from that of LECs in normal lung tissues. This idea is supported by a recent study using single cell sequence analysis, which revealed that the gene expression of LECs in LAM-affected lung tissues is distinct from that in normal lung tissues^19^. The functions of LECs may be altered in the pathological microenvironment of LAM lungs and still be conserved in vitro, with enhanced growth and migration potential and expression of VEGFR-3 and integrin α9.

VEGF-D acts as a ligand to VEGFR-2 or -3 on the cell surface of LECs and activates the PI3K-AKT pathway, which is crucial for lymphangiogenesis^20–24^. Because LAM cells produce VEGF-D, VEGF-D/VEGFR-2 or -3 signaling have been regarded as key mechanisms in LAM-associated lymphangiogenesis^2,3,11,12,25^. In the present study, the expression of VEGFR-3, but not VEGFR-2 was significantly higher in LAM-LECs (T) compared with control LECs. Furthermore, the PI3K-AKT signaling pathways were significantly enriched in LAM-LECs (T) and inhibition of VEGFR-3 activity suppressed the proliferation and migration of LAM-LECs (T). Similar to our results, a recent single cell sequence analysis of LAM-affected lung tissues unraveled that LECs highly express VEGFR-3, but not VEGFR-2^19^. Accordingly, VEGF-D/VEGFR-3 signaling rather than VEGF-D/VEGFR-2 signaling ought to be considered more predominant in LAM-associated lymphangiogenesis.

We also believe this is the first study that investigated the functional effects of VEGF-D on primary-cultured LECs isolated from human lungs. Though VEGF-D has been reported as a lymphangiogenic factor, we found that VEGF-D showed significant promotion only to migration, but had little effect on the proliferation of LECs. This is similar to the results in previous studies^26,27^. We think that VEGF-D appears to be more important in migration rather than proliferation of LECs. Because both proliferation and migration of LAM-LECs (T) were elevated, there may be other possible mechanisms which enhance proliferation of LECs in LAM. In this study, we found that VEGF-A markedly accelerated the proliferation of LECs, and that VEGF-C promoted the migration of LECs to almost the same degree as VEGF-D, both being suppressed by the inhibition of VEGFR-3 signaling. Considering these results, not only VEGF-D but also VEGF-A and -C likely have significant roles in the complex process of LAM-associated lymphanigiogenesis, including proliferation, sprouting and migration of LECs.

Integrin α9, a cell surface protein which forms a single heterodimer with β1, likely participates in tumor lymphangiogenesis because VEGF-C and D bind to integrin α9 to promote the migration of microvascular endothelial and tumor cells^28^. To date, however, no in vitro studies have reported the interaction of VEGF-D and integrin α9 using LECs. Our data revealed for the first time that VEGF-D-bound integrin α9 enhanced the migration of LAM-LECs (T) using the monoclonal anti-human integrin α9-neutralizing antibody. This result suggests that a new mechanism of LAM-associated lymphangiogenesis, VEGF-D/integrin α9 signaling, is also involved in LAM.

To explain the mechanisms for higher expression of VEGFR-3 and integrin α9 in LAM-LECs (T), the transcriptional factor PROX1, which plays a crucial role in differentiation of LECs, is likely the key factor^14,29,30^. Interestingly, both VEGFR-3 and integrin α9 are induced by PROX1 in the process of developmental lymphatics^31^. Several factors, such as homeobox transcription factor SOX18 (also known as SRY-Box Transcription Factor 18), the transcription factor hematopoietically expressed homeobox (HHEX), and Ets-2, have been reported as regulators of PROX1-induced lymphangiogenesis^32–34^. Preliminary result from our microarray data indicated expressions of PROX-1, HHEX, SOX-18 and Ets-2 were slightly increased in LAM-LECs (T) than control LECs (data not shown). However, the validation by RT-PCR showed no significant difference between two groups, possibly because of inter-individual variation of primary cells together with a small sample size. Accordingly, further study is required to elucidate the precise mechanism of upregulated VEGFR-3 and integrin α9 in LAM-LECs (T) with a larger sample size.

At present, mTOR inhibitors (e.g., rapamycin/sirolimus), are the first-line drugs for the treatment of LAM, which suppress abnormally activated mTOR signals in LAM cells^35^. Because LAM cells shed into lymphatic channels in LAM lesions by forming LAM cell clusters and disseminate^7^, anti-lymphangiogenic therapy is likely to be effective. Our data raise the possibility that both VEGFR-3 and integrin α9 can be therapeutic targets in LAM. The preceded studies reported that rapamycin exerted anti-lymphangiogenic effects by downregulating VEGFR-3 expression by LECs^36,37^. Additionally, taking the fact into consideration that both VEGFR-3-PI3K-AKT and integrin-FAK-PI3K-AKT signaling pathways converge at mTOR, rapamycin seems to have dual treatment targets in LAM pathobiology, e.g., LAM cells as well as LAM-associated LECs, to stabilize the disease course. The drugs directly acting to VEGFR-3 and integrin α9 pathways are likely to become therapeutic for LAM. Nintedanib is an inhibitor targeting multiple tyrosine kinases, including platelet-derived growth factor receptors, fibroblast growth factor receptors, and VEGFR-1, -2, and -3, and it is already approved for the treatment of idiopathic pulmonary fibrosis and non-small cell lung cancer^38–40^. Nintedanib is currently being tested in a Phase 2 clinical trial for LAM, with the expectation of suppressed proliferation of LAM cells and LAM-associated lymphangiogenesis (ClinicalTrials.gov, Identifier: NCT03062943). The anti-VEGFR-3 monoclonal antibody may also have a potential effect on LAM-associated lymphangiogenesis^41^. Conversely, for integrin α9, there have been no therapeutic applications for human diseases to date^42^. Further studies are warranted for the development of anti-integrin α9 therapy.

We acknowledge there were several limitations in this study. First, it is possible that the primary-cultured LECs that we used may not exactly reflect the characteristics of LECs in vivo, because the phenotype of LECs would be altered by various stimulants within in vitro environments after the isolation from lung tissues. Second, patient characteristics such as age, treatment, comorbidities, and respiratory status differed among individuals, which could have also influenced the signature of LECs. However, our model using primary-cultured LECs from patients is highly valuable because there is no cell line or animal model that precisely reflects the pathophysiology of LAM, due to its rarity.

In conclusion, we have successfully established the flow cytometry-based method to isolate and culture LECs from lung tissues. Moreover, we revealed the pathological phenotype of LAM-associated LECs which contributes to the excessive lymphangiogenesis in LAM.

## Materials and methods

### Patients and preparation of tissue samples

Tissue samples were prepared as we previously reported^15^. We obtained LAM-affected lung tissues from patients who underwent lung transplantation (*n* = 4) at the Department of Thoracic Surgery, Tohoku University Hospital (Sendai, Japan) or VATS for the treatment of pneumothorax (*n* = 3) at the Pneumothorax Research Center and Division of Thoracic Surgery, Nissan Tamagawa Hospital (Tokyo, Japan). Normal lung tissues were obtained from distal sites of tumors from patients who underwent lung resection for primary lung cancer (*n* = 4) at the Department of Thoracic Surgery, Juntendo University (Tokyo, Japan). Lung tissues were immediately immersed in tissue-preservation solution (Stem Survive-Lung; Kurabo, Osaka, Japan); they were preserved at 4℃ for several days or processed immediately to prepare single cell suspensions. This study was approved by the Institutional Review Board of the Juntendo Hospital (No. 19–132) and performed in accordance with the Declaration of Helsinki and the relevant guidelines and regulations. All participants gave written informed consent.

### Isolation and purification of LECs from lung cell suspensions

The preparation of single-cell suspensions from lung tissues is described in Supplementary Information. From lung cell suspension, we isolated CD45- lung cells using CD45 MicroBeads (Miltenyi Biotec, San Diego, USA) and an autoMACS Pro Separator (Miltenyi Biotec). Isolated CD45- lung cells were seeded onto collagen I-coated 6-well plates (Iwaki, Tokyo, Japan) or 10 cm dishes (Iwaki, Tokyo, Japan) according to the number of isolated cells, and cultured with a complete growth medium consisting of ECGM-MV2 (PromoCell, Heidelberg, Germany)/5% FBS (PromoCell)/100 U/ml of penicillin/streptomycin in a humidified incubator with a gas mixture of 21%O_2_, 5%CO_2_, and balance N_2_ at 37 °C until 70–80% confluence was achieved (usually reached in 7–10 days).

Next, we fractionated LECs from cultured CD45-lung cells using a fluorescence-activated cell sorting (FACS) Aria Fusion Cell Sorter (BD Biosciences, San Jose, USA) according to our previous protocol with some modification^15^. We used an Alexa Fluor 647-conjugated anti-human podoplanin antibody (303110, BioLegend, San Diego, USA), a fluorescein isothiocyanate (FITC)-conjugated anti-human CD31 antibody (303104, BioLegend) and isotype-matched control antibodies (BioLegend). To discriminate viable cells from dead cells, we used 7-amino actinomycin D (Thermo Fisher Scientific, Waltham, USA). Flow cytometry analyses were performed using the FlowJo v10 software package (BD Biosciences).

### Cell culture

Isolated LECs were seeded onto collagen I-coated plates (Iwaki) with ECGM-MV2 (PromoCell)/5% FBS/penicillin/streptomycin and cultured in a humidified incubator with a gas mixture of 5%O_2_, 5%CO_2_, and balance N_2_ at 37 °C. LECs were transferred to new dishes using 0.05% trypsin (Thermo Fisher Scientific) when they expanded to 70–80% confluence, or frozen aliquots were created using the cryopreservation solution Cellbanker 1 (Zenoaq, Koriyama, Japan) and stocked in liquid nitrogen. All the experiments described herein were performed on LECs from passages 2–8.

### Cell proliferation assay

A proliferation assay was performed using a Cell Counting Kit-8 ([CCK-8]; Dojindo Laboratories, mashiki, Japan), following the manufacturer’s protocol. LECs were seeded onto a 96-well collagen I-coated plate (FUJIFILM Wako Pure Chemical Corporation, Tokyo, Japan) at 4 × 10^3^ cells/well, incubated for 24 h, and followed by 6 h of serum-free starvation. After starvation, the serum-free media was replaced with ECBM (PromoCell)/5% FBS with or without growth factors; control samples were cultured with only ECBM/5% FBS, and other samples were separately cultured with recombinant human VEGF-A (10 ng/mL, R&D Systems, Minneapolis, USA), -C (50 ng/mL, R&D Systems), or -D (10 ng/mL, R&D Systems). The effects of MAZ51 (10 μM, Sigma-Aldrich, St. Louis, USA) and a monoclonal anti-human integrin α9-neutralizing antibody (50 μg/mL, MAB2078Z, Chemicon International, Temecula, USA) on proliferation of LECs were also tested.

Cell growth on Days 0, 1, 2 and 3 was assessed by examining absorbance (450 nm) of each well after 4 h from supplementing CCK-8 reagent into wells. The results were expressed as the absorbance at 450 nm measured by a microplate reader. Assays were performed in triplicate and data were expressed as means ± SD.

### Chamber migration assay

The mobility of LECs was analysed in 24-well plates (Corning) using Cell Culture Inserts (Corning) with a 8-µm pore size. The interior of the Cell Culture Inserts was coated with collagen I ([Cellmatrix Type I-C]; Nitta Gelatin, Osaka, Japan) following the manufacturer’s protocol. LECs were cultured in ECGM-MV2 with 5% FBS, and after 6 h of serum-free starvation, 1 × 10^5^ cells suspended in 200 µl of ECBM/0.5% FBS were seeded onto upper chambers. Lower chambers were filled with 700 µl of ECBM/0.5% FBS with or without growth factors. In the control condition, only ECBM/0.5% FBS was placed in lower chambers. In other groups, VEGF-A (10 ng/mL), -C (50 ng/mL) or -D (100 ng/mL) were added to ECBM/0.5% FBS as a chemoattractant. Cells were also dissociated by 0.05% trypsin/EDTA (Thermo Fisher Scientific), incubated with MAZ51 (10 μM) or a monoclonal anti-human integrin α9-neutralizing antibody (50 μg/mL), and seeded in the upper chamber.

After 4 h, the non-migrating cells on the surface of the upper chamber were removed with cotton swabs. The migrating cells at the bottom of the membrane were fixed using a Diff-Quik Stain Kit (Sysmex Corporation, Kobe, Japan) according to the manufacturer’s protocol. The number of migrated cells were counted on 4 randomly selected images under a bright-field light microscope. Data were expressed as means ± SD.

### cDNA microarray analysis

Total RNA from LAM-LECs (T) (*n* = 4) and control LECs (*n* = 2) was extracted with the RNeasy Plus Mini Kit (Qiagen, Hilden, Germany). Microarray analysis was performed using a SurePrint G3 Human GE v3 8 × 60 K Microarray (Agilent Technologies, Santa Clara, USA) according to standard protocols. The raw data were quantile normalized and log2-transformed for processing using GeneSpring GX14.9 software (Agilent Technologies). Pathway analyses for the genes in LAM-LECs (T) with greater than twofold expressions relative to the control LECs were determined using WikiPathways imported into GeneSpring GX14.9 software (Agilent Technologies).

### GSEA

Log-transformed cDNA microarray data were analysed using GSEA 4.0.3 software (Broad Institute, Cambridge, USA) according to published methods^43^. To disclose the genes associated with the ability of proliferation and migration in LAM-LECs (T) compared with control LECs, we analysed the expression of the gene sets of the GO terms “endothelial cell proliferation” and “endothelial cell migration” based on the Molecular Signatures Database (Broad Institute), as well as the LEC-specific gene signatures previously reported^14^. A nominal *p* value < 0.01 and a false discovery rate (FDR) < 0.25 were considered as statistically significant.

### qRT-PCR

Total RNAs were extracted using the RNeasy Plus Mini Kit (Qiagen) and cDNA was prepared using the SuperScript VILO cDNA Synthesis Kit (Thermo Fisher Scientific) according to manufacturer's protocols. qRT-PCR was performed using the StepOne Real-Time PCR System and PowerTrack SYBR Green Master Mix (Thermo Fisher Scientific) following the instructions. Data were examined by the 2-ΔΔCt method. Glyceraldehyde-3-phosphate dehydrogenase (*GAPDH*) was used as a housekeeping gene. Primers for qRT-PCR are listed in Supplementary Table S1.

### Flow cytometry analysis

The expression of VEGFR-2, VEGFR-3, and integrin α9 by isolated LECs were detected using the PE‑conjugated anti-human VEGFR-2 (359903, BioLegend), anti-human VEGFR-3 (356204, BioLegend), and anti-human integrin α9 antibodies (351605, BioLegend). An immunoglobulin isotype control (BioLegend) was used to distinguish specific from non‑specific bindings during flow cytometry. The expression levels of VEGFR-2, VEGFR-3, and integrin α9 were determined by the mean florescence intensity (MFI), using FlowJo v10 software (BD Biosciences).

### Immunofluorescence cytochemistry

Cytospun LECs were fixed with 8% paraformaldehyde, blocked, permeabilized and immmunostained^15^. We immunostained cells with: Anti-LYVE1 (clone Rabbit Polyclonal Antibody, Abcam, Cambrige, UK) as the primary antibody and Goat anti-Rabbit immunoglobulin (IgG Antibody, Alexa Fluor 594, Thermo Fisher Scientific) for detection of the primary antibody binding. 4′, 6-Diamidino-2-Phenylindole (DAPI; Vector Laboratories, Burlingame, CA) was used to stain nuclei. Immunofluorescence images were taken using Zeiss Axioplan2 Imaging (Zeiss, Oberkochen, Germany).

### Immunocytochemistry and immunohistochemistry

Cytospun LECs were fixed with 8% paraformaldehyde, blocked, and permeabilized. Frozen lung tissue blocks embedded with Tissue-Tek O.C.T. Compound (Sakura Finetek Japan, Tokyo, Japan) were utilized for immunohistochemistry and the Dako EnVision + System (Dako Cytomation, Produktionsvej, Denmark) was used to detect binding of the first antibody according to the manufacturer’s instructions. We used 7 primary antibodies as shown in Supplementary Table S2. 3,3′-diaminobenzidine tetrahydrochloride (DAB) or Fast-Red was used as the chromogen.

### Statistical analyses

Statistical analyses were performed with GraphPad Prism 7.0 software (GraphPad Software, La Jolla, USA). For direct comparisons between 2 groups, significance was assessed using unpaired Student’s t-tests. In experiments with multiple groups, significance was analysed using one-way analysis of variance (ANOVA) followed by Tukey’s test. A *p* value < 0.05 was considered statistically significant. All quantitative data are represented as mean ± SD.

## Supplementary Information

Supplementary Information

## Data Availability

The datasets generated and analyzed in this study are available from the corresponding author upon reasonable request.
