# Supplementary material for: Isolation and characterisation of lymphatic endothelial cells from lung tissues affected by lymphangioleiomyomatosis
Source: Sci Rep. 2021 Apr 16;11:8406. doi: 10.1038/s41598-021-88064-3 (PMC8052438; doi:10.1038/s41598-021-88064-3)
Supplement: Supplementary file 1 — Supplementary Information [file 41598_2021_88064_MOESM1_ESM.pdf]

Supplementary Information

Isolation and Characterisation of Lymphatic Endothelial Cells from Lung Tissues Affected by Lymphangioleiomyomatosis

Koichi Nishino[1, 2]*, Yasuhiro Yoshimatsu[3, 4], Tomoki Muramatsu[5], Yasuhito Sekimoto[1, 2], Keiko Mitani[1, 2], Etsuko Kobayashi[1, 2], Shouichi Okamoto[1, 2], Hiroki Ebana[1, 2, 6, 7], Yoshinori Okada[8], Masatoshi Kurihara[2, 6], Kenji Suzuki[9], Johji Inazawa[5], Kazuhisa Takahashi[1], Tetsuro Watabe[3], and Kuniaki Seyama[1, 2]

[1] Division of Respiratory Medicine, Juntendo University Faculty of Medicine and Graduate School of Medicine

[2] Study Group for Pneumothorax and Cystic Lung Diseases

[3] Department of Biochemistry, Graduate School of Medical and Dental Sciences, Tokyo Medical and Dental University

[4] Division of Pharmacology, Graduate School of Medical and Dental Sciences, Niigata University

[5] Department of Molecular Cytogenetics, Medical Research Institute, Tokyo Medical and Dental University

[6] Pneumothorax Research Center and Division of Thoracic Surgery, Nissan Tamagawa Hospital

[7] Department of Thoracic Surgery, Tokyo Metropolitan Bokutoh Hospital

[8] Department of Thoracic Surgery, Institute of Development, Aging and Cancer, Tohoku University

[9] Department of General Thoracic Surgery, Juntendo University School of Medicine

***Corresponding author:**

Koichi Nishino

Division of Respiratory Medicine, Juntendo University Faculty of Medicine and Graduate School of Medicine, 3-1-3 Hongo; Bunkyoku, Tokyo 113-8431, Japan

Tel: +81-3-5802-1063, Fax: +81-3-5802-1617, E-mail: k-nishino@juntendo.ac.jp

Supplementary Materials and Methods

Preparation of single-cell suspensions from lung tissues

Human lung tissues were processed to prepare single-cell suspensions as previously described[1]. Briefly, lung tissues were cut into 2 to 3 g of specimen and put into 50-ml conical tubes containing neutral protease (2.0 U/mL Dispase II, Roche Applied Science, Penzberg, Germany), Collagenase/Dispase (1 mg/mL, Roche Applied Science), and deoxyribonuclease (0.1 mg/mL DNase I, Sigma-Aldrich, St. Louis, USA) in 50-ml conical tubes. The tissues were roughly minced by scissors in the conical tubes and incubated for 30 min with shaking (1 Hz). Next, the tissues were minced with scissors again and reincubated for another 60 min with shaking. Thereafter, the tissues were passed through an 18-gauge needle 5 times.

Subsequently, we added 10 ml of Dulbecco’s Modified Eagle Medium (Thermo Fisher Scientific, Waltham, USA) containing 10% fetal bovine serum and 100 U/ml of penicillin/streptomycin, and filtered the mixture through a Falcon 100-µm Cell Strainer (Corning, Corning, USA) 2 times. After centrifugation at 1,500 revolutions/min (rpm) for 5 min, we discarded the supernatant and added 5 ml of Red Blood Cell Lysis Buffer (Roche Applied Science) to the cell pellet and continued incubation for 3 min at room temperature. After incubation, we added 15 ml of culture medium and centrifuged at

1,500 rpm for 5 min. We repeated the lysis step, followed by resuspension in 10 ml of culture medium and filtering 2 times through a Falcon 40-μm Cell Strainer (Corning).

Supplementary Table S1. List of primers for qRT-PCR

Target Gene		Primer Sequence
GAPDH	Forward	TGATGACATCAAGAAGGTGGTGAAG
	Reverse	TCCTTGGAGGCCATGTGGGCCAT
VEGFR-2	Forward	CCTCCCCCGCATCACAT
	Reverse	GCTCGTTGGCGCACTCTT
VEGFR-3	Forward	TCTGCTACAGCTTCCAGGTGG
	Reverse	GCAGCCAGGTCTCTGTGGAT
ITGA9	Forward	CAAAGGCATCGGCAAGGTTT
	Reverse	TCCCCATTCAGGTCAACTGC

Supplementary Table S2. List of antibodies for immunohistochemistry

Antibody	Clone	Company	Dilution
Anti-human Calretinin	DAK-Calret-1	Dako A/S	1:50
Anti-human CK5/6	D5/16B4	Dako A/S	1:100
Anti-human podoplanin	D2-40	Dako A/S	1:200
Anti-human Prox-1	Rabbit Poly	AngioBio	1:1000
Anti-human LYVE-1	Rabbit Poly	Abcam	1:100
Anti-human VEGFR-3	Goat Poly	R&D Systems	1:50
Anti-human integrin α9β1	2Q954	Santa Cruz Biotechnology	1:100

Supplementary Table S3. **Up-regulated genes in LAM-LECs (T) regarding focal adhesion and the PI3K-AKT signaling pathway**

**Probe Name**	**Fold change**	**Gene symbol**	**Description**
A_33_P3304668	7.510	*COL1A1*	Collagen, Type I, Alpha 1
A_24_P334300	6.351	*FGF12*	Fibroblast Growth Factor 12
A_23_P217319	5.947	*FGF13*	Fibroblast Growth Factor 13
A_24_P759477	3.895	*ITGB8*	Integrin, Beta 8
A_33_P3335725	3.874	*INSR*	Insulin Receptor
A_33_P3307267	3.425	*VWF*	Von Willebrand Factor
A_23_P65240	3.201	*COL4A1*	Collagen, Type IV, Alpha 1
A_23_P201636	2.603	*LAMC2*	Laminin, Gamma 2
A_23_P205031	2.521	*COL4A2*	Collagen, Type IV, Alpha 2
A_23_P60079	2.501	*ANGPT2*	Angiopoietin 2
A_32_P208403	2.426	*GNG2*	Guanine Nucleotide Binding Protein, Gamma 2
A_24_P124349	2.370	*PDGFD*	Platelet Derived Growth Factor D
A_23_P252193	2.363	*ITGA9*	Integrin, Alpha 9
A_24_P277934	2.355	*COL1A2*	Collagen, Type I, Alpha
A_24_P59667	2.306	*JAK3*	Janus Kinase 3
A_23_P153897	2.290	*GNG7*	Guanine Nucleotide Binding Protein, Gamma 7
A_23_P113005	2.248	*EFNA1*	Ephrina1
A_33_P3323959	2.244	*RELN*	Reelin
A_24_P339944	2.135	*PDGFB*	Platelet Derived Growth Factor Beta Polypeptide
A_33_P3212274	2.108	*F2R*	Coagulation Factor II Receptor
A_23_P156708	2.090	*TNXB*	Tenascin XB
A_23_P160968	2.048	*LAMC2*	Laminin, Gamma 2

Among the genes regarding cell adhesion and the PI3K-AKT -mTOR pathway, the up-regulated genes in LAM-LECs (T) with a fold change greater than 2 are listed in descending order, according to fold change calculated by GeneSpring software (Agilent Technologies, Santa Clara, USA).

Supplementary Table S4. Up-regulated genes in control LECs regarding cell adhesion and the PI3K-AKT pathway

Probe Name	Fold change	Gene symbol	Description
A_23_P217379	22.356	*COL4A6*	Collagen, Type IV, Alpha 6
A_23_P45365	5.735	*COL4A5*	Collagen, Type IV, Alpha 5
A_33_P3305790	3.740	*NOS3*	Nitric Oxide Synthase 3
A_23_P404494	3.511	*IL7R*	Interleukin 7 Receptor
A_33_P3377364	3.382	*ITGB4*	Integrin, Beta 4
A_23_P338534	3.273	*HIF3A*	Hypoxia Inducible Factor 3, Alpha Subunit
A_23_P83818	3.024	*COL5A1*	Collagen, Type V, Alpha 1
A_23_P109269	3.004	*LAMA5*	Laminin, Alpha 5
A_23_P142187	2.800	*HIF3A*	Hypoxia Inducible Factor 3, Alpha Subunit
A_21_P0000025	2.706	*NOS3*	Nitric Oxide Synthase 3
A_33_P3629678	2.689	*COL5A1*	Collagen, Type V, Alpha 1
A_33_P3351944	2.655	*EGFR*	Epidermal Growth Factor Receptor
A_33_P3338121	2.576	*LAMB3*	Laminin, Beta 3
A_23_P89780	2.486	*LAMA3*	Laminin, Alpha 3
A_23_P166633	2.084	*ITGB5*	Integrin, Beta 5

Among the genes regarding cell adhesion and the PI3K- AKT-mTOR pathway, the upregulated genes in control LECs with a fold change greater than 2 are listed in descending order, according to fold change calculated by GeneSpring software (Agilent Technologies).

Supplementary Table S5. Leading-edge genes of LEC-specific genes enriched in LAM-LECs (T)

Probe	Description	Rank Metric Score
DSP	Desmoplakin	3.610
ALDH1A1	Aldehyde Dehydrogenase 1 Family, Member A1	2.802
HLA-DPB1	Major Histocompatibility Complex, Class II, DP Beta 1	2.645
MAF	V-Maf Avian Musculoaponeurotic Fibrosarcoma Oncogene Homolog	2.398
DCLK1	Doublecortin-like Kinase 1	2.265
RBP1	Retinol Binding Protein 1, Cellular	2.101
PEG10	Paternally Expressed 10	2.099
SEPP1	Selenoprotein P, Plasma, 1	2.096
CLEC4M	C-Type Lectin Domain Family 4, Member M	2.094
FABP4	Fatty Acid Binding Protein 4, Adipocyte	1.987
UNC5B	Unc-5 Homolog B (C. Elegans)	1.915
RGS16	Regulator of G-Protein Signaling 16	1.914
SOCS2	Suppressor of Cytokine Signaling 2	1.894
CH25H	Cholesterol 25-Hydroxylase	1.887
SLC26A4	Solute Carrier Family 26 (Anion Exchanger), Member 4	1.876
LAMP3	Lysosomal-Associated Membrane Protein 3	1.742
TIMP3	TIMP Metallopeptidase Inhibitor 3	1.664
RAMP3	Receptor (G Protein-Coupled) Activity Modifying Protein 3	1.610
ARID5B	AT Rich Interactive Domain 5B (MRF1-Like)	1.606
CDKN1C	Cyclin-Dependent Kinase Inhibitor 1C (P57, Kip2)	1.560
PCSK6	Proprotein Convertase Subtilisin/Kexin Type 6	1.552
MAOA	Monoamine Oxidase A	1.465
HOXD10	Homeobox D10	1.451
GPRC5B	G Protein-Coupled Receptor, Class C, Group 5, Member B	1.443
IGFBP2	Insulin-Like Growth Factor Binding Protein 2, 36kDa	1.430

SPRY1	Sprouty Homolog 1, Antagonist of FGF Signaling (Drosophila)	1.353
ANGPT2	Angiopoietin 2	1.322
IL7	Interleukin 7	1.256
NDRG1	N-Myc Downstream Regulated 1	1.240
ITGA9	Integrin, Alpha 9	1.240
RAMP2	Receptor (G Protein-Coupled) Activity Modifying Protein 2	1.209
PDPN	Podoplanin	1.164
RELN	Reelin	1.139
LMO2	LIM Domain Only 2 (Rhombotin-like 1)	1.049
CSRP2	Cysteine and Glycine-Rich Protein 2	1.042
CALCRL	Calcitonin Receptor-like	1.016
PRKCZ	Protein Kinase C, Zeta	1.008
SMAGP	Small Cell Adhesion Glycoprotein	1.007
LIPA	Lipase A, Lysosomal Acid, Cholesterol Esterase	0.945
ISG20	Interferon- Stimulated Exonuclease Gene 20kDa	0.894
MRC1	Mannose Receptor, C Type 1	0.874
PDE8A	Phosphodiesterase 8A	0.842
OLFML2A	Olfactomedin-Like 2A	0.823
CEACAM1	Carcinoembryonic Antigen-Related Cell Adhesion Molecule 1 (Biliary Glycoprotein)	0.777
TUBA4A	Tubulin, Alpha 4a	0.776
PAQR3	Progestin and Adipoq Receptor Family Member III	0.750
NR2F1	Nuclear Receptor Subfamily 2, Group F, Member 1	0.745
MEF2C	Myocyte Enhancer Factor 2C	0.692
SLC39A14	Solute Carrier Family 39 (Zinc Transporter), Member 14	0.681
PPP1R2	Protein Phosphatase 1, Regulatory (Inhibitor) Subunit 2	0.677
RMND5A	Required for Meiotic Nuclear Division 5 Homolog A (S. Cerevisiae)	0.676
KIAA0101	Kiaa0101	0.674
ADD3	Adducin 3 (Gamma)	0.673
BTBD3	BTB (POZ) Domain Containing 3	0.660
TK1	Thymidine Kinase 1, Soluble	0.657

PPAP2A	Phosphatidic Acid Phosphatase Type 2A	0.632
GCH1	GTP Cyclohydrolase 1	0.605
CTDSPL	CTD (Carboxy-Terminal Domain, RNA Polymerase II, Polypeptide A) Small Phosphatase-like	0.540
MYBL2	V-Myb Avian Myeloblastosis Viral Oncogene Homolog-like 2	0.515
REEP1	Receptor Accessory Protein 1	0.514
AURKB	Aurora Kinase B	0.505
DUSP5	Dual Specificity Phosphatase 5	0.498
LYN	LYN Proto-Oncogene, Src Family Tyrosine Kinase	0.495
ACSL3	Acyl-Coa Synthetase Long-Chain Family Member 3	0.484
GINS1	GINS Complex Subunit 1 (Psf1 Homolog)	0.471
TBX1	T-Box 1	0.465
RBPMS	RNA Binding Protein with Multiple Splicing	0.465
HIST1H4C	Histone Cluster 1, H4c	0.455
LMNB1	Lamin B1	0.408
FLT4	Fms-Related Tyrosine Kinase 4	0.404
CKS1B	CDC28 Protein Kinase Regulatory Subunit 1B	0.403
CCNA2	Cyclin A2	0.401
CETP	Cholesteryl Ester Transfer Protein, Plasma	0.399

LEC-specific genes enriched in LAM-LECs (T) compared to control LECs are listed in descending order, according to the rank metric score calculated by Gene Set Enrichment Analysis software (Broad Institute, Cambridge, USA).

References

1 Ando, K. *et al.* Isolation of individual cellular components from lung tissues of patients with lymphangioleiomyomatosis. *Am J Physiol Lung Cell Mol Physiol* **310**, L899-908, doi:10.1152/ajplung.00365.2015 (2016).
